# Medical Association of Georgia

**Published:** 1884-05

**Authors:** 


### Editorial.

### MEDICAL ASSOCIATION OF GEORGIA.

The thirty-fifth annual session of the Medical Association of Georgia, was held on the 16th, 17th and 18th of April, in the city of Macon, within a stones throw of the building in which it was organized in the spring of 1849. It was sad to note that only two or three of those who were present and assisted in the organization at its first meeting were in attendance, the great majority having long since passed away, and many others still alive, have been driven from active duty in the profession by age and infirmities.

A striking feature of the personel of the recent meeting was the marked predominance of the younger members of the profession, which we regard as a favorable omen for the future of the Association. As the older members pass away, or fail to attend the meeting from causes above suggested, the younger members present themselves in increased numbers, ready to assume the responsibilities, the labors and honors of the Association, and with that energy and vim characteristic of the youth of the present age, will push the Association to increased membership and usefulness.

Another hopeful sign of the future of the Association is the fact that with the single exception of the meeting held in Atlanta, the late meeting in Macon was the largest in attendance of any held for the past many years, and as will be seen from a synopsis of the proceedings of the meeting in another page, the papers presented and read either by title, synopsis or entire, were greater in number, interest, and of a decidedly higher order than at any meeting for the past twenty years. Another encouraging feature preceptible was, that the members present appeared to feel a deep and lively interest in the future of the Association—a determination to go to work with renewed energy and leave nothing undone to make it the pride, and an honor to the profession of the State.

No one who is familiar with the condition of the profession of the State in 1849, and will review the work of the Association since its organization, but will readily admit the great good the profession has derived from its annual meetings. While it is true that in times past some of the meetings of the Association were attended by strife, bickerings and bad blood, among its members, in which scenes were enacted not very complimentary to the organization, since the adoption of the new constitution transferring the disciplining of members with other business matters of the Association to a Board of Censors, much of the friction that formerly resulted in unpleasant scenes as above referred to are prevented, so that the mass of the Association is confined to the presentation and discussion of scientific and practical subjects, while the Board of Censors are attending to the business of the Association.

We feel that the Association has just commenced a new era, and from this time her course will be onward and upward, that if her older devotees who have for so many years labored for her success continue their labors with unabated energy, and that the younger members of the profession who are now swelling her ranks, will continue for a time the energy and vim displayed at the recent meeting in Macon, in a few years all will be surprised at the success attained, and the great good to the profession that will eminate from the organization.

